# Trends and Disparities in Technology Use and Glycemic Control in Type 1 Diabetes

**DOI:** 10.1001/jamanetworkopen.2025.26353

**Published:** 2025-08-11

**Authors:** Michael Fang, Yunwen Xu, Shoshana H. Ballew, Josef Coresh, Justin B. Echouffo-Tcheugui, Elizabeth Selvin, Jung-Im Shin

**Affiliations:** 1 Department of Epidemiology, Johns Hopkins Bloomberg School of Public Health, Baltimore, Maryland; 2 Department of Population Health, Optimal Aging Institute and Division of Epidemiology, New York University Grossman School of Medicine, New York, New York; 3 Department of Medicine, Division of Endocrinology, Diabetes and Metabolism, Johns Hopkins School of Medicine, Baltimore, Maryland

## Abstract

**Importance:**

Characterizing population-level changes in type 1 diabetes (T1D) management can inform public health policies and interventions.

**Objective:**

To characterize trends and disparities in glycemic control and use of diabetes technology among US youths and adults with T1D.

**Design, Setting, and Participants:**

This serial, cross-sectional analysis used the Optum Labs Data Warehouse, a national, deidentified database of electronic health records, to identify US youths (aged <18 years) and adults (aged ≥18 years) with T1D. Data were obtained from records from January 1, 2009, to December 31, 2023.

**Exposures:**

Calendar years divided into 3-year study periods from 2009 to 2011 to 2021 to 2023.

**Main Outcomes and Measures:**

Glycemic control (mean hemoglobin A_1c_ level, <7%) and use of diabetes technology (continuous glucose monitoring systems and/or insulin pumps) were defined using laboratory data and prescriptions, procedures, and diagnoses codes from electronic health records.

**Results:**

A total of 186 590 participants with T1D was identified (mean [SD] age, 40 [19] years; 96 766 [52%] male; 12 493 [7%] Hispanic, 2819 [2%] non-Hispanic Asian, 21 459 [12%] non-Hispanic Black, and 141 847 [76%] non-Hispanic White). Of these, 26 853 participants were youths (mean [SD] age, 12 [4] years; 14 060 [52%] male; 19 822 [74%] non-Hispanic White) and 159 737 were adults (mean [SD] age, 45 [16] years; 82 706 [52%] male; 122 025 [76%] non-Hispanic White). From the 2009-2011 to 2021-2023 study periods, the prevalence of glycemic control (mean hemoglobin A_1c_ level <7%) increased from 7% (95% CI, 7%-8%) to 19% (95% CI, 19%-20%) in youths (*P* < .001 for trend) and 21% (95% CI, 21%-22%) to 28% (95% CI, 28%-29%) in adults (*P* < .001 for trend). During this same period, there was a substantial increase in the percentage of patients using continuous glucose monitoring (4% to 82% for youths; 5% to 57% for adults), insulin pumps (16% to 50% for youths; 11% to 29% for adults), and both devices concurrently (1% to 47% for youths; 1% to 22% for adults) (*P* < .001 for trend for all). The prevalence of glycemic control and use of diabetes technology were lowest in Hispanic, non-Hispanic Black, and Medicaid-insured youths and adults, and differences persisted or increased over time.

**Conclusions and Relevance:**

In this cross-sectional study, there was a rapid increase in the use of diabetes technology and notable improvements in glycemic control among youths and adults with T1D during the past 15 years. Nonetheless, the prevalence of glycemic control remained low, and racial, ethnic, and socioeconomic differences grew over time.

## Key Points

**Question:**

Has glycemic control changed during the past 15 years among individuals with type 1 diabetes (T1D)?

**Findings:**

In this cross-sectional study of 186 590 individuals with T1D, the prevalence of glycemic control increased in US youths (7% to 19%) and adults (21% to 28%) between the 2009-2011 and 2021-2023 study periods. Patients who were Hispanic or non-Hispanic Black and insured by Medicaid had the lowest prevalence of glycemic control, and differences across ethnicity, race, and insurance status grew over time.

**Meaning:**

These findings suggest that glycemic control has improved significantly in US youths and adults with T1D during the past 15 years, but overall prevalence remains low.

## Teaser

This cross-sectional study characterizes trends and disparities in the attainment of glycemic control and use of diabetes technologies among US youths and adults with type 1 diabetes.

## Introduction

Glycemic control is essential for preventing complications and premature mortality in persons with type 1 diabetes (T1D).^1,2,3^ During the past 2 decades, major advances in diabetes technologies have enabled patients with T1D to reach glycemic targets more safely and effectively.^4,5,6,7,8^ Devices including continuous glucose monitoring (CGM) systems and insulin pumps are now recommended for all persons with T1D.^9^ However, rates of technology use and glycemic control remain poorly characterized in the general population with T1D.

Emerging studies have consistently documented racial, ethnic, and socioeconomic disparities in the management of T1D.^10,11,12,13,14,15,16,17^ However, these have included a small number of patients or have focused on non-Hispanic White patients in high-income groups who receive care from specialists at academic medical centers.^16,17^ There are few population-based studies of T1D in the US.^18^ Large, representative, contemporary data can broaden the generalizability of prior studies and inform efforts to address health disparities in T1D.

Our objective was to characterize trends and disparities in the attainment of glycemic control and use of diabetes technologies (CGM and/or insulin pumps) among US youths and adults with T1D. To accomplish these goals, we analyzed 15 years of data (January 1, 2009, to December 31, 2023) in a large, diverse, national cohort of patients with T1D.

## Methods

### Data Source and Study Population

We analyzed deidentified electronic health record (EHR) data in the Optum Labs Data Warehouse (OLDW). The OLDW database contains longitudinal health information on more than 200 million patients from 60 different health systems throughout the US.^19,20^ The patient population in the OLDW represents a mixture of ages and geographical regions across the US, allowing for a comprehensive examination of health disparities. EHR data are collected within individual health systems and harmonized into a single standardized database. The OLDW database includes sociodemographic information, prescription orders (eg, devices for diabetes management), health care encounters, and laboratory results. This study was approved by the Johns Hopkins Bloomberg School of Public Health Institutional Review Board, which waived the need for informed consent for use of deidentified data. We followed the Strengthening the Reporting of Observational Studies in Epidemiology (STROBE) reporting guideline.

We restricted our study population to youths (aged <18 years) and adults (aged ≥18 years) with T1D in the OLDW between January 1, 2007, and December 31, 2023 (N = 282 875) (study flowchart is found in eFigure 1 in Supplement 1). To ensure study patients were active members, we excluded patients who were not continuously enrolled in OLDW for at least 12 months following their T1D diagnosis (n = 41 652). We additionally excluded patients with no measurement of hemoglobin A_1c_ (HbA_1c_) level during the study period (n = 54 633). Our final analytic sample consisted of 186 590 patients (26 853 youth and 159 737 adults) with T1D.

### T1D Ascertainment

We ascertained T1D status using a modified version of the Klompas algorithm (eFigure 2 in Supplement 1).^21,22,23^ This algorithm has been previously validated in different health systems and is widely used in EHR-based studies of T1D.^21,22,23,24,25,26,27^ The Klompas algorithm determines T1D status using a 2-step approach. First, we identified patients with any diabetes, defined as (1) at least 1 code for diabetes in inpatient encounters from the *International Classification of Diseases, Ninth Revision* (*ICD-9*) or *International Statistical Classification of Diseases and Related Health Problems, Tenth Revision* (*ICD-10*); (2) at least 2 *ICD-9* or *ICD-10* codes for diabetes in outpatient encounters within 2 years (codes are found in eTable 1 in Supplement 1); (3) a prescription for 1 or more medications to lower glucose levels (excluding metformin); or (4) 2 or more elevated laboratory test results for diabetes (fasting glucose level ≥126 mg/dL, nonfasting glucose level ≥200 mg/dL [to convert to mmol/L, multiply by 0.0555], or HbA_1c_ level ≥6.5%) within 2 years.^28^ Among persons with diabetes, we classified patients as having T1D if they had an insulin prescription and met any of the following criteria: (1) at least 50% of diabetes diagnosis codes were for T1D and a prescription of glucagon; (2) at least 50% of diabetes diagnosis codes were for T1D and no prescriptions for medications to lower glucose levels other than insulin or metformin; (3) at least 1 positive autoantibody test result; or (4) a negative C-peptide test result (<0.8 ng/mL [to convert to nmol/L, multiply by 0.331]).

### Study Periods

We divided the OLDW data into 3-year periods (January 1, 2009, to December 31, 2011; January 1, 2012, to December 31, 2014; January 1, 2015, to December 31, 2017; January 1, 2018, to December 31, 2020; and January 1, 2021, to December 31, 2023) to improve the precision and stability of our estimates. For each study period, we included all patients with T1D who had at least 1 HbA_1c_ measurement available (eFigure 1 in Supplement 1).

### Glycemic Control and Technology Use

For each patient, we calculated mean HbA_1c_ levels using all available measurements within each 3-year study period. In our main analyses, we defined glycemic control as a mean HbA_1c_ level of less than 7.0%.^29^ In sensitivity analyses, we used 3 alternative cut points (<6.5%, <7.5%, and <8.0%) to define glycemic control.^29,30^

Consistent with prior studies, we identified the use of CGM and insulin pumps using National Drug Codes in prescriptions, procedure, and diagnoses codes from EHR data (codes are found in eTable 2 in Supplement 1). In each study period, we examined the percentage of patients who used CGM, insulin pumps, or both devices concurrently.

### Sociodemographic Measures

Sociodemographic information (age, sex, race, ethnicity, and insurance type) was extracted from the EHR data. We categorized patients into age groups (2-12, 13-17, 18-44, 45-64, and ≥65 years) based on their age at first HbA_1c_ measurement during each study period. Race and ethnicity (Hispanic, non-Hispanic Asian, non-Hispanic Black, non-Hispanic White, and other or unknown) data were all collected by health systems within the OLDW.

### Statistical Analysis

We described characteristics of youths and adults with T1D at the time of their first HbA_1c_ measurement. We estimated the prevalence of glycemic control and use of diabetes technologies in youths and adults in each study period (2009-2011, 2012-2014, 2015-2017, 2018-2020, and 2021-2023). We calculated exact 95% CIs using the binomial distribution. We conducted these analyses overall and by age group (2-12, 13-17, 18-44, 45-64, and ≥65 years), ethnicity and race (Hispanic, non-Hispanic Black, and non-Hispanic White; non-Hispanic Asian was excluded owing to small sample size), and insurance type (commercial, Medicaid, or Medicare).

We tested for trends in glycemic control and use of diabetes technology with logistic mixed-effect regression models. For these analyses, we modeled the midpoint of each study period as a continuous linear measure. We also included a random intercept for participants to account for the inclusion of the same patients across multiple study periods.^31^

In sensitivity analyses, we repeated our analyses (1) using alternative cut points for glycemic control and (2) using a more specific definition of T1D based only on laboratory test results (antibody positive or low C-peptide level). We also calculated changes in mean HbA_1c_ levels in youths and adults with T1D.

All analyses were conducted using Stata, version 16.1 (StataCorp LLC), or R, version 4.2.1 (R Foundation for Statistical Computing). Two-sided *P* < .05 was considered statistically significant.

## Results

### Study Population

The OLDW included 241 223 youths and adults with T1D from 2009 to 2023. Among these patients, 186 590 (77%) had at least 1 measurement of HbA_1c_ level available and were included in the analysis (mean [SD] age, 40 [19] years; 89 824 [48%] female and 96 766 [52%] male; 12 493 [7%] Hispanic, 2819 [2%] non-Hispanic Asian, 21 459 [12%] non-Hispanic Black, and 141 847 [76%] non-Hispanic White) (eFigure 1 in Supplement 1). Most patients (126 496 [68%]) were included in multiple study periods (eTable 3 in Supplement 1). Patients with who were older, were non-Hispanic Black, and had no insurance information were more likely to have missing HbA_1c_ data (eTable 4 in Supplement 1).

The study sample included 26 853 youths (mean [SD] age, 12 [4] years; 12 793 [48.1%] female and 14 060 [52%] male; 2687 [10%] Hispanic, 312 [1%] non-Hispanic Asian, 2521 [9%] non-Hispanic Black, and 19 822 [74%] non-Hispanic White) and 159 737 adults (mean [SD] age, 45 [16] years; 77 031 [48%] female and 82 706 [52%] male; 9806 [6%] Hispanic, 2507 [2%] non-Hispanic Asian, 18 938 [12%] non-Hispanic Black, and 122 025 [76%] non-Hispanic White) with T1D. Approximately two-thirds of all patients had commercial insurance. Race and ethnicity data were unavailable for 7972 (4%) patients in the study population (eTable 5 in Supplement 1).

Between the 2009-2011 and 2021-2023 study periods, the age distribution among youths with T1D remained unchanged, while the proportion who identified as Hispanic or non-Hispanic Black increased (eTable 6 in Supplement 1). Among adults with T1D, the proportion of patients who were 65 years or older, identified as non-Hispanic Black, or were insured under Medicaid increased.

### Trends in Glycemic Control

**Figure 1.**
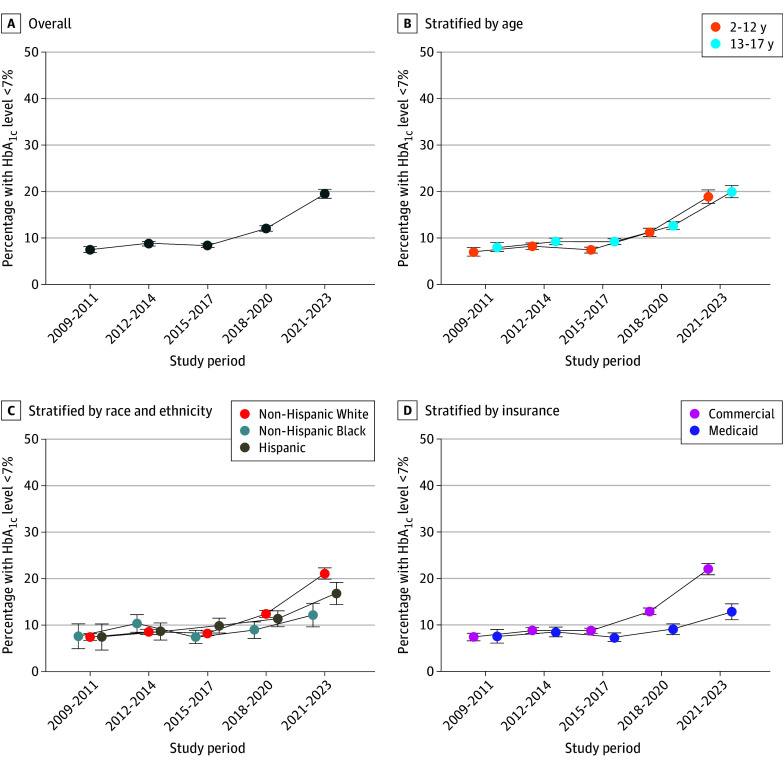
Trends in the Prevalence of Glycemic Control Among Youths With Type 1 Diabetes Includes 26 853 participants. Glycemic control is defined as hemoglobin A_1c_ (HbA_1c_) level of less than 7%. Error bars indicate the 95% CIs for each estimate.

The prevalence of glycemic control (HbA_1c_ level <7%) among youths with T1D was unchanged from the 2009-2011 to 2014-2017 study periods (approximately 7% [95% CI, 7%-8%] to 9% [95% CI, 8%-9%]) before increasing to 19% (95% CI, 19%-20%) in the 2021-2023 period (*P* < .001 for trend) (Figure 1A). Glycemic control significantly increased for all youth subgroups except for patients who were non-Hispanic Black (7% [95% CI, 5%-10%] to 12% [95% CI, 10%-15%]; *P* = .71 for trend). Differences in glycemic control by race, ethnicity, and insurance type increased after the 2018-2020 period. During the 2021-2023 period, 21% (95% CI, 20%-22%) of non-Hispanic White youths had glycemic control, compared with 17% (95% CI, 14%-19%) of Hispanic and 12% (95% CI, 10%-15%) of non-Hispanic Black youths. Glycemic control was also higher for youths with commercial vs Medicaid insurance (22% [95% CI, 21%-23%] vs 13% [95% CI, 11%-15%]) in the 2021-2023 period (Figure 1B-D).

**Figure 2.**
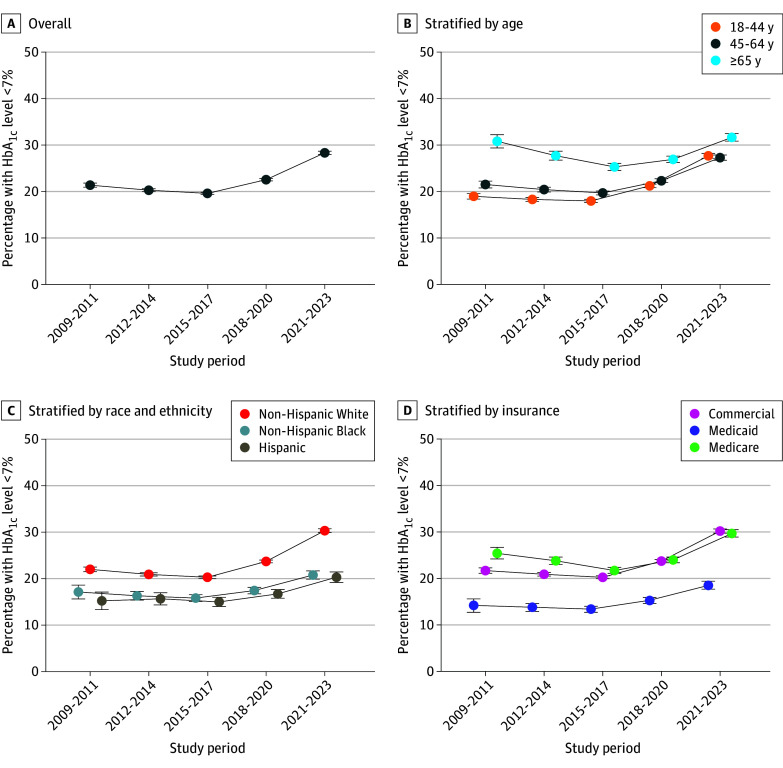
Trends in the Prevalence of Glycemic Control Among Adults With Type 1 Diabetes Includes 159 737 participants. Glycemic control is defined as hemoglobin A_1c_ (HbA_1c_) level of less than 7%. Error bars indicate the 95% CIs for each estimate.

Among adults with T1D, the prevalence of glycemic control was stable from the 2009-2011 (21% [95% CI, 21%-22%]) to the 2014-2017 (20% [95% CI, 20%-21%]) study periods but rose to 28% (95% CI, 28%-29%) during the 2021-2023 study period (*P* < .001 for trend) (Figure 2A). Glycemic control increased for all subgroups after the 2015-2017 period (*P* < .001 for trend for all groups), but changes were largest among non-Hispanic White and commercially insured adults. In the 2021-2023 period, the prevalence of glycemic control was 30% (95% CI, 30%-31%) in non-Hispanic White adults compared with 20% (95% CI, 19%-21%) in Hispanic and 21% (95% CI, 20%-22%) in non-Hispanic Black patients. Glycemic control was higher in adults with commercial vs Medicaid insurance (30% [95% CI, 30%-30%] vs 19% [95% CI, 18%-19%]) (Figure 2B-D).

Trends were similar using different cut points to define glycemic control (eFigure 3 in Supplement 1) and when defining T1D only using autoantibody and C-peptide measurements (eFigure 4 in Supplement 1). From the 2009-2011 to 2021-2023 study periods, mean (SD) HbA_1c_ level decreased from 8.9% (1.5%) to 8.3% (1.7%) in youths and 8.2% (1.7%) to 8.0% (1.7%) in adults with T1D, with changes occurring after the 2015-2017 period (eTable 7 in Supplement 1).

### Trends in Use of Diabetes Technology 

**Figure 3.**
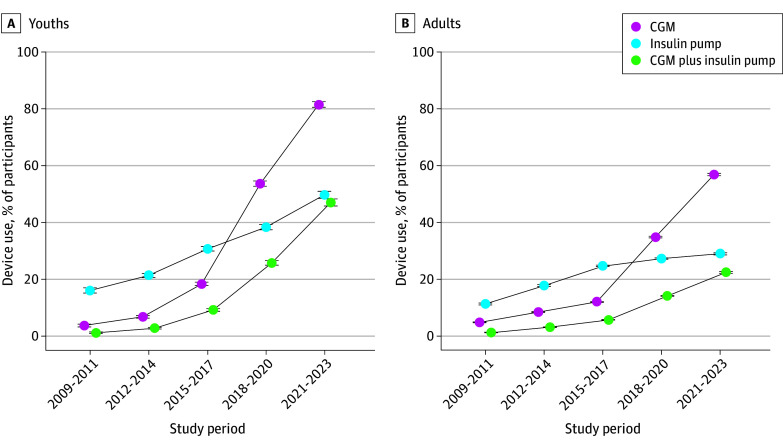
Trends in the Use of Continuous Glucose Monitoring (CGM) and Insulin Pumps Among Patients With Type 1 Diabetes Error bars indicate the 95% CIs for each estimate.

From the 2009-2011 to 2021-2023 study periods, there was a substantial increase in the use of CGM (4% [95% CI, 3%-4%] to 82% [95% CI, 81%-83%]; *P* < .001 for trend) and insulin pumps (16% [95% CI, 15%-17%] to 50% [95% CI, 81%-83%]; *P* < .001 for trend) among youths with T1D (Figure 3A). The percentage of youths using both devices concurrently also grew, from 1% (95% CI, 1%-1%) to 47% (95% CI, 46%-48%) (*P* < .001 for trend).

**Figure 4.**
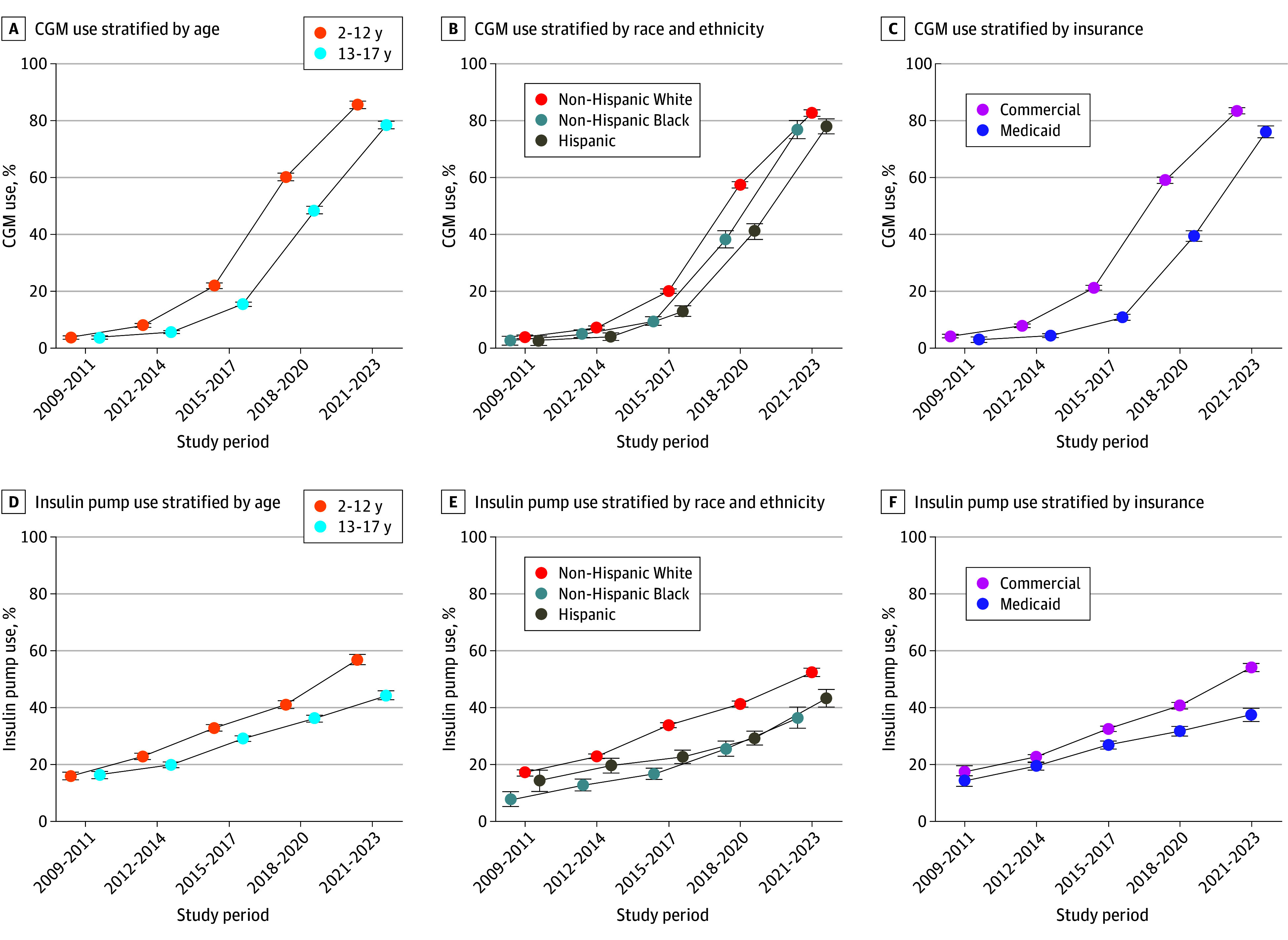
Trends in the Use of Continuous Glucose Monitoring (CGM) and Insulin Pumps Among Youths With Type 1 Diabetes Error bars indicate the 95% CIs for each estimate.

In the 2021-2023 study period, CGM and insulin pump use were highest in youths younger than 13 years (86% [95% CI, 84%-87%] for CGM; 57% [95% CI, 55%-59%] for insulin pump), those who were non-Hispanic White (83% [95% CI, 82%-84%] for CGM; 52% [95% CI, 51%-54%] for insulin pump), or those who were commercially insured (83% [95% CI, 82%-84%] for CGM; 54% [95% CI, 53%-56%] for insulin pump). Differences in diabetes technology use across populations emerged in the 2015-2017 study period (*P* < .001 for trend for all groups) (Figure 4). Differences in CGM use across age, race, ethnicity, and insurance type narrowed from the 2018-2020 to 2021-2023 periods (Figure 4A-C). In contrast, differences in insulin pump use across age, race, ethnicity, and insurance type increased over time (Figure 4D-F).

**Figure 5.**
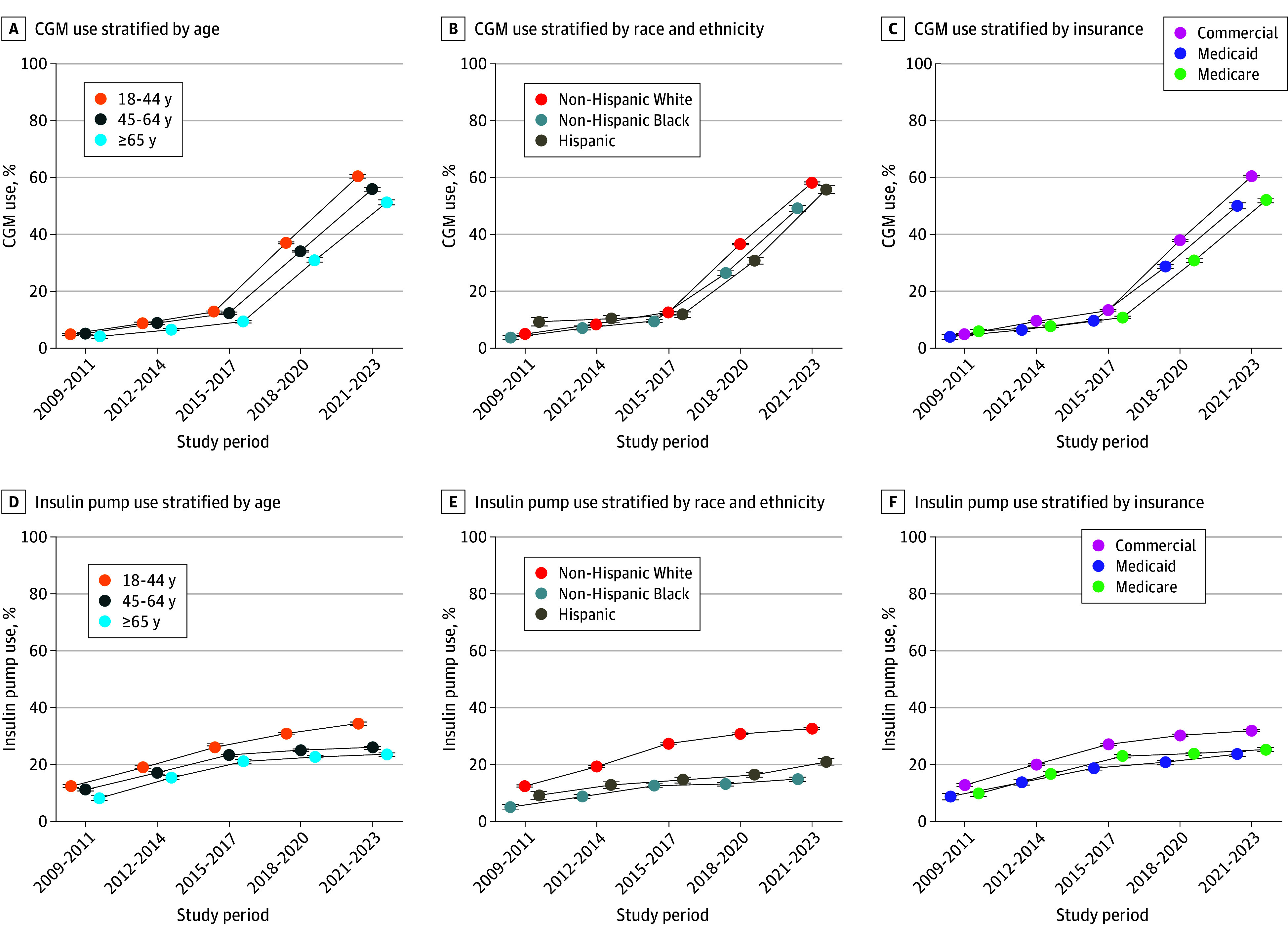
Trends in the Use of Continuous Glucose Monitor (CGM) and Insulin Pumps Among Adults With Type 1 Diabetes Error bars indicate the 95% CIs for each estimate.

In adults with T1D, there was an increase in the use of CGM (5% [95% CI, 5%-5%] to 57% [95% CI, 57%-57%]; *P* < .001 for trend), insulin pumps (11% [95% CI, 11%-12%] to 29% [95% CI, 29%-29%]; *P* < .001 for trend), and both devices concurrently (1% [95% CI, 1%-1%] to 22% [95% CI, 22%-23%]; *P* < .001 for trend) from the 2009-2011 to 2021-2023 study periods (Figure 3B). Adults with T1D who were younger, non-Hispanic White, and commercially insured were more likely to use CGM (Figure 5A-C) or insulin pumps (Figure 5D-F); these differences were unchanged over time.

In analyses examining the joint association of race and ethnicity with insurance status, non-Hispanic White youths and adults with commercial insurance had the highest prevalence of use of diabetes technology, while non-Hispanic Black and Hispanic patients who had Medicaid insurance had the lowest prevalence (eTables 8 and 9 in Supplement 1).

## Discussion

From the 2009-2011 to 2021-2023 study periods, there were notable improvements in glycemic control among US youth and adults with T1D. However, the overall prevalence of glycemic control remained low. Approximately 19% of youths and 28% of adults with T1D currently have HbA_1c_ levels less than 7%.

Our results update and extend prior US studies on glycemic control in T1D. Consistent with our findings, data from the Type 1 Diabetes Exchange Registry found that glycemic control was unchanged between the 2010-2012 to 2016-2018 periods, with 17% of youths and 21% of adults reaching glycemic targets.^16^ The SEARCH for Diabetes in Youth Study found small increases in mean HbA_1c_ levels from the 2002-2007 to 2014-2019 periods in youths and young adults with T1D.^32^ The present analyses, which include more contemporary data in a large, national study sample, highlight a potential turning point in T1D management, with encouraging improvements in glycemic control occurring since the late 2010s.

The growing use of diabetes technologies may have contributed to improved glycemic management in patients with T1D. In particular, trends in glycemic control followed patterns of CGM use, with both increasing after 2017. The rising use of CGM in the late 2010s is likely attributable to several factors. First, insurance coverage expanded significantly during this period, broadening CGM access for persons with T1D.^8,33^ Medicare, for instance, began covering CGM for beneficiaries in 2017.^34^ Second, CGM devices were rapidly integrated into clinical guidelines for diabetes management.^35,36^ In 2018, the American Diabetes Association first recommended CGM for all children with T1D.^37^ In 2021, the American Diabetes Association expanded this recommendation to include all persons treated with insulin, regardless of age or diabetes type.^38^ Third, CGM accuracy, functionality, and ease of use continued to improve significantly over time.^39^

Expanding the use of diabetes technologies may further promote glycemic control, particularly for adults. CGM and insulin pumps are now recommended for all persons with T1D, regardless of age.^9^ However, in our study, only 57% of adults were using CGM and 29% were using insulin pumps during the 2021-2023 period. Our results are consistent with prior studies and highlight the underuse of novel technologies in adults with T1D.^16^ Additional opportunities for individualized patient and clinician education can help optimize the use of technologies. In our analyses, the increase in CGM and insulin pump use substantially outpaced gains in glycemic control, suggesting that these technologies may not improve management for many patients.

There was a large increase in the concurrent use of CGM and insulin pumps, reaching 47% in youths and 22% in adults by the 2021-2023 period. Pairing CGM and insulin pumps to automate insulin delivery is associated with larger improvements in glycemic control and quality of life compared with use of these devices alone.^40^ However, these “closed-loop” systems require 2 devices and may be challenging to access for many patients.^41^ Guidelines also emphasize a “rigorous, comprehensive, consistent, and structured education curriculum” for patients using automated insulin delivery, since systems differ significantly from traditional approaches (eg, multiple daily insulin injections).^42^ Enhancing coverage and patient education may help promote the adoption of these novel systems.

Consistent with prior studies,^16,17^ we found that racial, ethnic, and socioeconomic disparities in CGM use among youths increased from 2015 to 2020. However, differences narrowed substantially during the 2021-2023 period, and approximately 75% to 80% of all youths with T1D were using CGM. Changes in insurance coverage likely contributed to these patterns. For example, Medicaid coverage for CGM in youths with T1D expanded from 35 states in 2019 to 41 states in 2022.^8^ Nonetheless, we found that other disparities in use of diabetes technology persisted over time. In youths, the uptake of the insulin pump increased faster among non-Hispanic White and commercial insured youths. In adults, racial, ethnic, and socioeconomic differences in the use of both CGM and insulin pumps largely persisted throughout the study period. Thus, despite some progress, broad access to diabetes technology remains an ongoing challenge, especially among adults with T1D.

Similar to prior research,^16,43,44^ we found large differences in glycemic control across race, ethnicity, and insurance status that widened over time. These disparities may be partly related to differential uptake of diabetes technologies. Additionally, social factors, including access to treatment and care, socioeconomic resources, and clinician bias, also influence glycemic control and likely disparities in T1D management.^45,46^ Reducing disparities will require broadening access to novel technologies, along with interventions that address social determinants of health.

### Strengths and Limitations

Our study has important strengths. This is, to our knowledge, one of the largest studies of T1D in the US. We included patients from different demographic backgrounds throughout the US. We had more than 15 years of contemporary clinical data and analyzed trends across the life span (age range, 2-88 years). The large sample size allowed for precise estimates in subgroups.

Our study also has several limitations. First, the ascertainment of T1D was based on an algorithm that used a combination of medications, laboratory tests, and diagnostic codes.^21^ Nonetheless, this algorithm has been validated and shown to be highly specific across different settings.^22,23^ Moreover, results were consistent when defining T1D only using laboratory measures (positive autoantibody or negative C-peptide test results). Second, while OLDW is geographically diverse, the sample may not be representative of all US persons with T1D. Patients who were older and non-Hispanic Black had higher rates of missing HbA_1c_ data compared with individuals who were younger and non-Hispanic White. However, the characteristics of our study participants were consistent with other large US studies of adults and children with T1D, including recent nationally representative analyses.^47,48^ Third, HbA_1c_ levels were only measured in patients who engaged with the health care system and received routine care. However, this issue may be minimized because patients with T1D are typically highly engaged with the health care system owing to their absolute need for insulin. Indeed, 77.4% of persons with T1D in OLDW had HbA_1c_ levels assessed during the study period, with small differences across sociodemographic characteristics.

Fourth, our analyses separately examined trends in glycemic control and technology use at a population level. Careful interpretation is needed for determining the causal association of these two at an individual level. Fifth, our analyses were crude and did not adjust for potential confounding factors that may be underlying differences in glycemic control or technology use across age, race, ethnicity, and insurance status. Sixth, we were unable to determine whether patients were using closed-loop insulin delivery systems (CGM, insulin pumps, and an algorithm that automatically adjusts insulin delivery) vs open-loop sensor augmented insulin pump therapy (without an algorithm). Seventh, the collection of race and ethnicity data was not standardized, and there may be some differences across health systems. Eighth, EHR data may not fully capture diabetes technology use. For example, some patients may obtain diabetes technology devices through medical suppliers without an active prescription.

## Conclusions

This cross-sectional study found that during the past 15 years there was a rapid increase in use of diabetes technology and notable improvements in glycemic control among youths and adults with T1D. Nonetheless, the prevalence of glycemic control remains low, and disparities across race, ethnicity, and socioeconomic status have persisted or worsened over time.

## Notes

Supplement 1. **eFigure 1.** Flowchart of Study Enrollment **eFigure 2.** Study Design **eFigure 3.** Trends in the Prevalence of Glycemic Control Using Different Cut Points in Youth and Adults With Type 1 Diabetes From 2009-2011 to 2021-2023 **eFigure 4.** Trends in the Prevalence of Glycemic Control in Youths and Adults With Laboratory-Confirmed Type 1 Diabetes From 2009-2011 to 2021-2023 **eTable 1.** *International Classification of Diseases* (*ICD*) Codes for Diabetes **eTable 2.** Codes Used for Identifying Insulin Pump and Continuous Glucose Monitor Use **eTable 3.** Number of Study Periods in Which Patients Were Included, by Age **eTable 4.** Characteristics of Patients With Type 1 Diabetes With and Without HbA_1c_ **eTable 5.** Characteristics of Youth and Adults With Type 1 Diabetes at the First Measurement of HbA_1c_ in the Study **eTable 6.** Characteristics of Youth and Adults With Type 1 Diabetes at the First Measurement of HbA_1c_ by Year **eTable 7.** Mean Hemoglobin A_1c_ in Youth and Adults With Type 1 Diabetes From 2009-2011 to 2021-2023 **eTable 8.** Trends in Use of Continuous Glucose Monitoring and Insulin Pumps in Youths With Type 1 Diabetes From 2009-2011 to 2021-2023 by Race, Ethnicity, and Insurance Status **eTable 9.** Trends in Use of Continuous Glucose Monitoring and Insulin Pumps in Adults With Type 1 Diabetes From 2009-2011 to 2021-2023 by Race, Ethnicity, and Insurance Status

Supplement 2.
Data Sharing Statement
