# Supplementary material for: Trends and Disparities in Technology Use and Glycemic Control in Type 1 Diabetes
Source: JAMA Netw Open. 2025 Aug 11;8(8):e2526353. doi: 10.1001/jamanetworkopen.2025.26353 (PMC12340658; doi:10.1001/jamanetworkopen.2025.26353)
Supplement: Supplement 1. — eFigure 1. Flowchart of Study Enrollment eFigure 2. Study Design eFigure 3. Trends in the Prevalence of Glycemic Control Using Different Cut Points in Youth and Adults With Type 1 Diabetes From 2009-2011 to 2021-2023 eFigure 4. Trends in the Prevalence of Glycemic Control in Youths and Adults With Laboratory-Confirmed Type 1 Diabetes From 2009-2011 to 2021-2023 eTable 1. International Classification of Diseases (ICD) Codes for Diabetes eTable 2. Codes Used for Identifying Insulin Pump and Continuous Glucose Monitor Use eTable 3. Number of Study Periods in Which Patients Were Included, by Age eTable 4. Characteristics of Patients With Type 1 Diabetes With and Without HbA1c eTable 5. Characteristics of Youth and Adults With Type 1 Diabetes at the First Measurement of HbA1c in the Study eTable 6. Characteristics of Youth and Adults With Type 1 Diabetes at the First Measurement of HbA1c by Year eTable 7. Mean Hemoglobin A1c in Youth and Adults With Type 1 Diabetes From 2009-2011 to 2021-2023 eTable 8. Trends in Use of Continuous Glucose Monitoring and Insulin Pumps in Youths With Type 1 Diabetes From 2009-2011 to 2021-2023 by Race, Ethnicity, and Insurance Status eTable 9. Trends in Use of Continuous Glucose Monitoring and Insulin Pumps in Adults With Type 1 Diabetes From 2009-2011 to 2021-2023 by Race, Ethnicity, and Insurance Status [file jamanetwopen-e2526353-s001.pdf]

Supplementary Online Content

Fang M, Xu Y, Ballew SH, et al. Trends and disparities in technology use and glycemic control in type 1 diabetes. *JAMA Netw Open.* 2025;8(8):e2526353. doi:10.1001/jamanetworkopen.2025.26353

eFigure 1. Flowchart of Study Enrollment

eFigure 2. Study Design

eFigure 3. Trends in the Prevalence of Glycemic Control Using Different Cut Points in Youth and Adults With Type 1 Diabetes From 2009-2011 to 2021-2023

eFigure 4. Trends in the Prevalence of Glycemic Control in Youths and Adults With Laboratory-Confirmed Type 1 Diabetes From 2009-2011 to 2021-2023

eTable 1. *International Classification of Diseases* (*ICD*) Codes for Diabetes

eTable 2. Codes Used for Identifying Insulin Pump and Continuous Glucose Monitor Use

eTable 3. Number of Study Periods in Which Patients Were Included, by Age

eTable 4. Characteristics of Patients With Type 1 Diabetes With and Without HbA_{1c}

eTable 5. Characteristics of Youth and Adults With Type 1 Diabetes at the First Measurement of HbA_{1c} in the Study

eTable 6. Characteristics of Youth and Adults With Type 1 Diabetes at the First Measurement of HbA_{1c} by Year

eTable 7. Mean Hemoglobin A_{1c} in Youth and Adults With Type 1 Diabetes From 2009-2011 to 2021-2023

eTable 8. Trends in Use of Continuous Glucose Monitoring and Insulin Pumps in Youths With Type 1 Diabetes From 2009-2011 to 2021-2023 by Race, Ethnicity, and Insurance Status

eTable 9. Trends in Use of Continuous Glucose Monitoring and Insulin Pumps in Adults With Type 1 Diabetes From 2009-2011 to 2021-2023 by Race, Ethnicity, and Insurance Status

This supplementary material has been provided by the authors to give readers additional information about their work.

© 2025 Fang M et al. *JAMA Network Open*.

eFigure 1. Flowchart of Study Enrollment

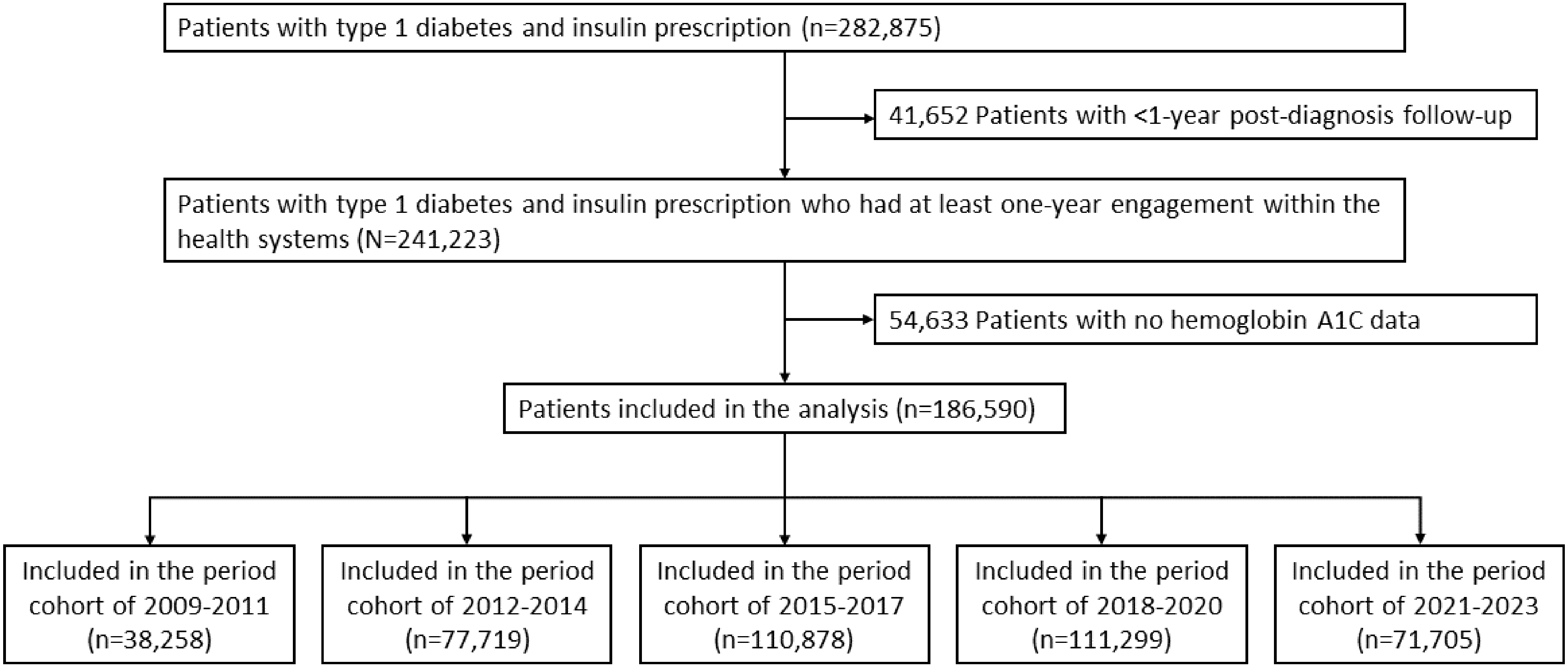

Abbreviations: OLDW = Optum Labs Data Warehouse

© 2025 Fang M et al. *JAMA Network Open*.

eFigure 2. Study Design

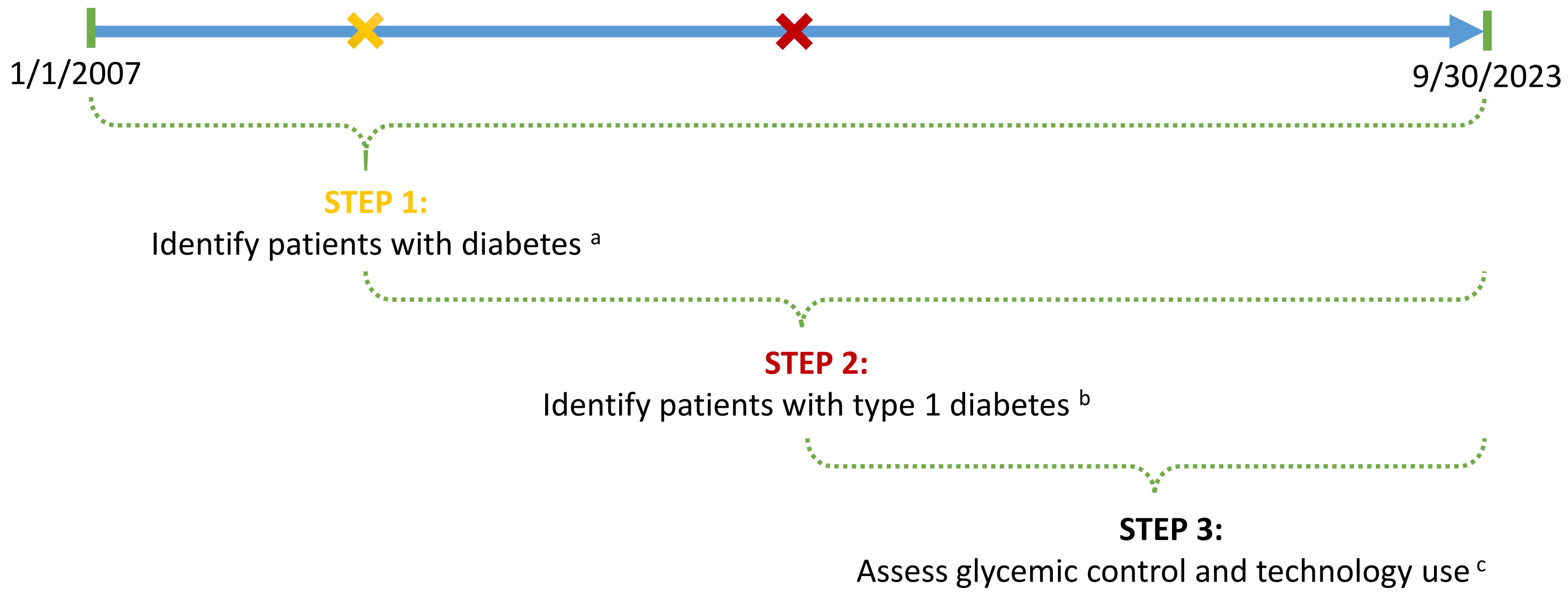

Note: Brackets indicate the time period considered for each step of the analysis. The yellow "X" indicates the date when a hypothetical patient meets the criteria for having any diabetes (Step 1). The red "X" indicates the date when a hypothetical patient meets the criteria for having type 1 diabetes (Step 2).

[a] Using data from the entire study period, patients were classified as having diabetes if they met any of the following criteria: (1) diagnosis codes in one inpatient record or two outpatient records within 2 years, or (2) one or more prescription for diabetes medications excluding metformin, or (3) two or more lab test results (fasting glucose ≥126 mg/dL, non-fasting glucose ≥200 mg/dL, or hemoglobin A1c ≥6.5%) within 2 years.

[b] Using data following the diagnosis of diabetes, patients were classified as having type 1 diabetes if they met any of the following criteria: (1) ratio of type 1 to type 2 diabetes diagnosis codes >0.5 and a prescription of glucagon; (2) ratio of type 1 to type 2 diabetes diagnosis codes >0.5 and no prescriptions for glucose-lowering medications other than insulin or metformin; (3) at least one positive autoantibody test; (4) c-peptide levels <0.8 ng/mL.

[c] Patients with type 1 diabetes were required to have at least 12 months of continuous engagement within a health system to be included in the assessment of glycemic control of technology use.

© 2025 Fang M et al. *JAMA Network Open*.

eFigure 3. Trends in the Prevalence of Glycemic Control Using Different Cut Points in Youth and Adults With Type 1 Diabetes From 2009-2011 to 2021-2023

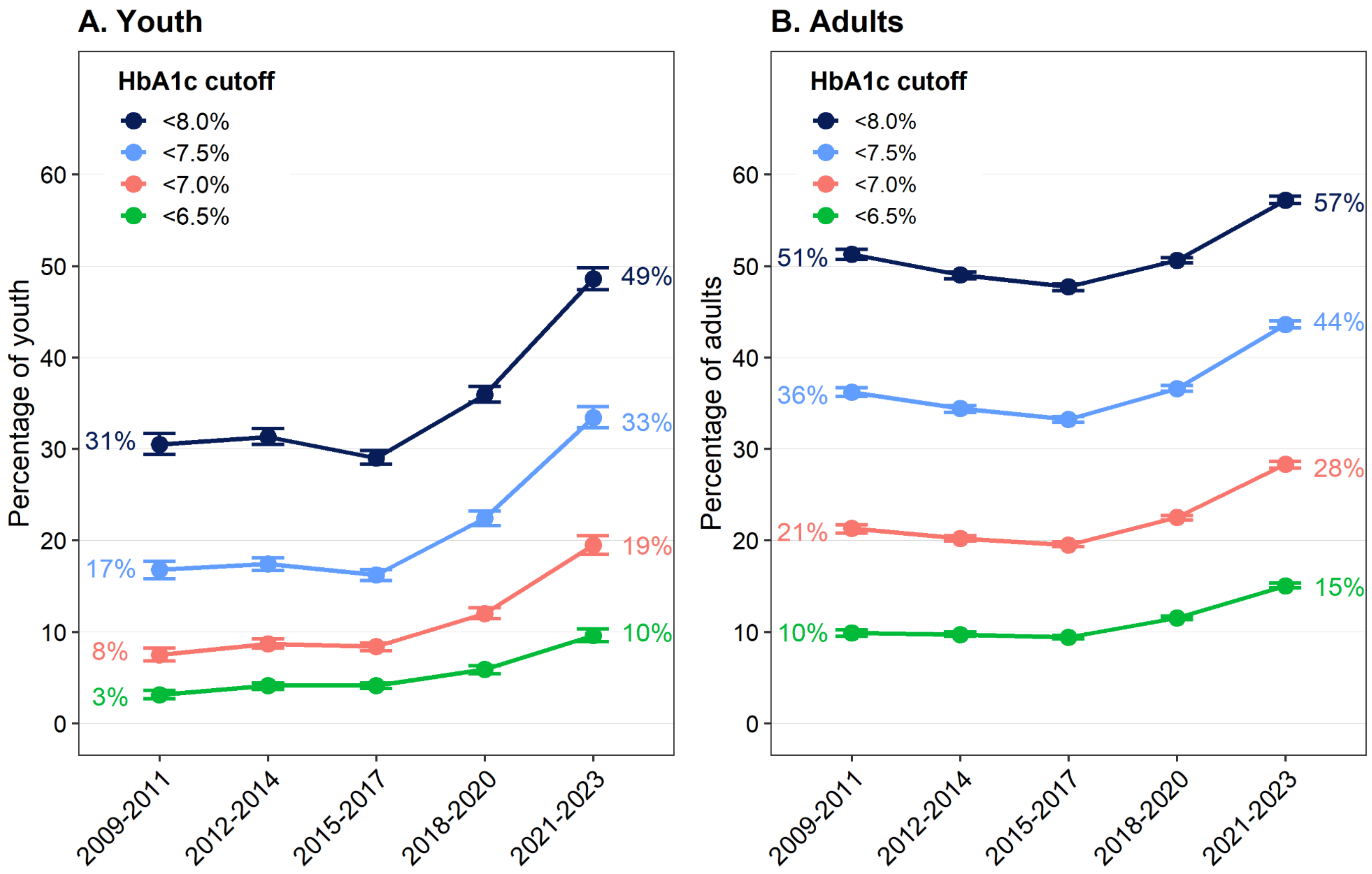

Note: Error bars indicate the 95% confidence intervals for each estimate.

© 2025 Fang M et al. *JAMA Network Open*.

eFigure 4. Trends in the Prevalence of Glycemic Control in Youths and Adults With Laboratory-Confirmed Type 1 Diabetes From 2009-2011 to 2021-2023

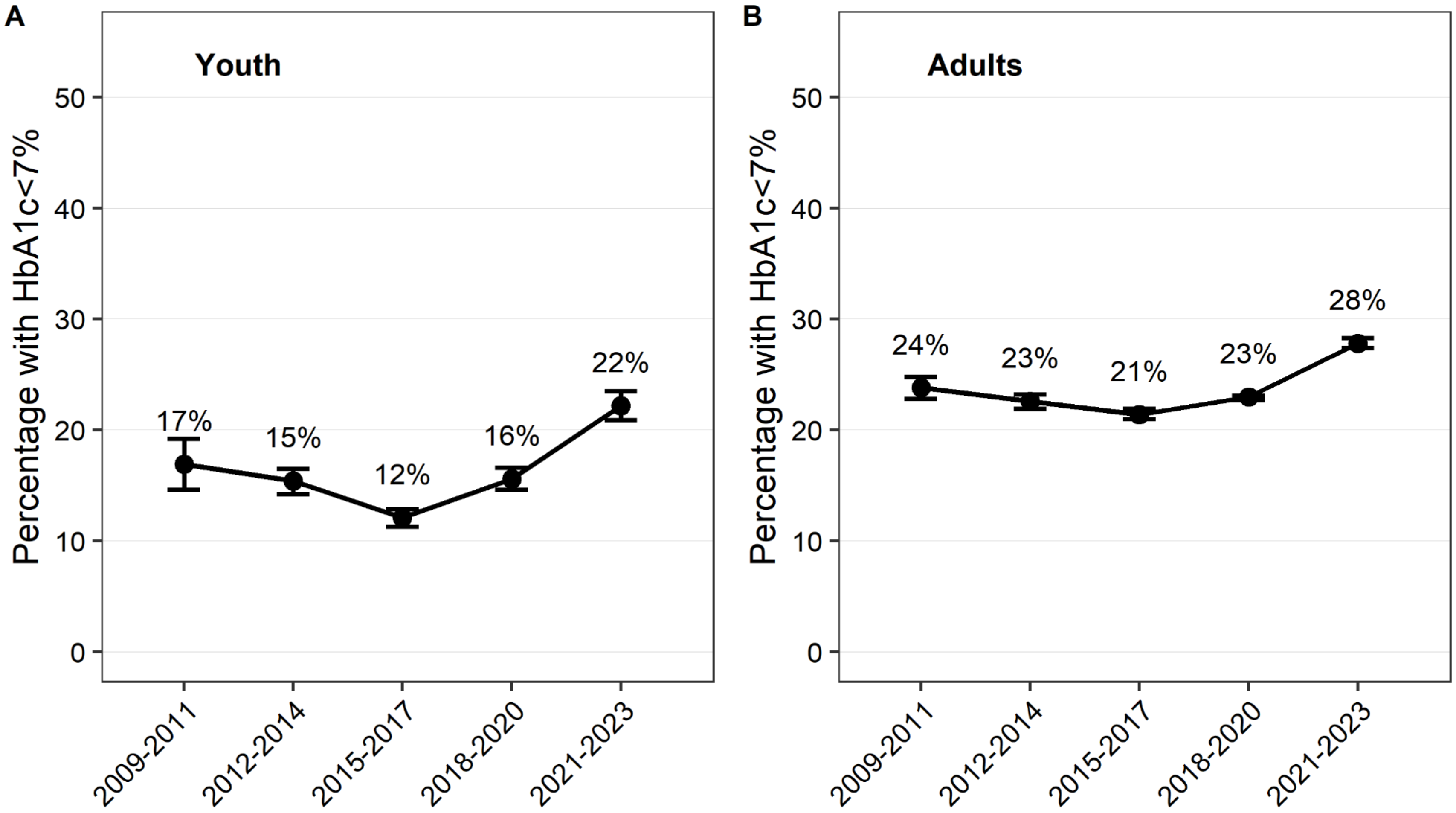

Note: Type 1 diabetes was defined as having a positive autoantibody or a negative c-peptide (<0.8 ng/mL) test result. Error bars indicate the 95% confidence intervals for each estimate.

© 2025 Fang M et al. *JAMA Network Open*.

eTable 1. *International Classification of Diseases* (*ICD*) Codes for Diabetes

	**ICD 9**	**ICD 10**
Type 1 diabetes	250.x1, 250.x3	E10
Type 2 diabetes	250.x0, 250.x2	E11
Others	249.x (secondary diabetes), 357.2 (neuropathy in diabetes), 362.0x (diabetic retinopathy), 366.41 (diabetic cataract)	E08–E09 (secondary diabetes), E13 (other specified diabetes mellitus)

© 2025 Fang M et al. *JAMA Network Open*.

eTable 2. Codes Used for Identifying Insulin Pump and Continuous Glucose Monitor Use

**Insulin pump**	Diagnosis codes: Z9641, V4585, Z4681, V6545, V5391<br><br>Procedure codes: A4230, A4231, A4232, A4224, A4225, E0784, E0787, S9145, S9353, A4224, J1811, J1813, J1817
**Continuous glucose monitor**	Procedure codes: 95249, 95250, 95251, 99091, 0446T, 0447T, 0448T, A9276, A9277, A9278, S1030, S1031 K0553, K0554, E1399, A9999, A4239, E2103<br><br>The type of device is identified using the National Drug Code (NDC), which is a unique 10-digit number that serves as a universal product identifier for on all nonprescription and prescription medication packages in the United States.<br>NDC codes: 43169080040, 43169095568, 63000028585, 63000028677, 63000028678, 76300000260, 76300000610, 76300000701, 76300001101, 76300007202, 76300010001, 76300023982, 76300031501, 76300040001, 76300070001, 76300070501, 76300070601, 76300090001, 76300700610, 76300731501, 76300740001, 76300770001, 76300790001, 43169070405, 76300017962 (Guardian products); 08627006011, 08627006031, 08627006021, 08627003011, 08627003031, 08627003021, 08627005011, 08627005031, 08627005021, 08627002011, 08627002031, 08627002021, 08627001301, 08627001101, 08627005104, 08627008011, 08627008031, 08627008021, 08627001401, 08627009111, 08627005303, 08627001601, 08627009011, 08627073101, 08627007701 (Dexcom products); 57599000019, 57599000021, 57599000101, 57599000200, 57599080300, 57599080000, 57599082000, 57599081800, 57599084400, 57599083500 (Freestyle Libre products).
**Insulin pump**	Diagnosis codes: Z9641, V4585, Z4681, V6545, V5391<br><br>Procedure codes: A4230, A4231, A4232, A4224, A4225, E0784, E0787, S9145, S9353, A4224, J1811, J1813, J1817

© 2025 Fang M et al. *JAMA Network Open*.

**Continuous glucose monitor**	Procedure codes: 95249, 95250, 95251, 99091, 0446T, 0447T, 0448T, A9276, A9277, A9278, S1030, S1031 K0553, K0554, E1399, A9999, A4239, E2103<br><br>The type of device is identified using the National Drug Code (NDC), which is a unique 10-digit number that serves as a universal product identifier for on all nonprescription and prescription medication packages in the United States.<br>NDC codes: 43169080040, 43169095568, 63000028585, 63000028677, 63000028678, 76300000260, 76300000610, 76300000701, 76300001101, 76300007202, 76300010001, 76300023982, 76300031501, 76300040001, 76300070001, 76300070501, 76300070601, 76300090001, 76300700610, 76300731501, 76300740001, 76300770001, 76300790001, 43169070405, 76300017962 (Guardian products); 08627006011, 08627006031, 08627006021, 08627003011, 08627003031, 08627003021, 08627005011, 08627005031, 08627005021, 08627002011, 08627002031, 08627002021, 08627001301, 08627001101, 08627005104, 08627008011, 08627008031, 08627008021, 08627001401, 08627009111, 08627005303, 08627001601, 08627009011, 08627073101, 08627007701 (Dexcom products); 57599000019, 57599000021, 57599000101, 57599000200, 57599080300, 57599080000, 57599082000, 57599081800, 57599084400, 57599083500 (Freestyle Libre products).

© 2025 Fang M et al. *JAMA Network Open*.

eTable 3. Number of Study Periods in Which Patients Were Included, by Age

	Median (IQI)	Number of study periods				
		1	2	3	4	5
Youth with type 1 diabetes (unique individuals, n= 26,853)	2 [1, 2]	11,010	9,997	4,624	1,107	115
Adults with type 1 diabetes (unique individuals, n=159,737)	2 [1, 3]	58,414	54,935	32,861	16,457	5,457

Note: Among 186,590 patients with type 1 diabetes who had HbA1c measurements, 126,496 (67.8%) individuals were included in multiple study periods.

© 2025 Fang M et al. *JAMA Network Open*.

eTable 4. Characteristics of Patients With Type 1 Diabetes With and Without HbA_{1c}

	Patients with type 1 diabetes and insulin use N=241,223	Without HbA1C N=54,633 (22.6%)	With HbA1C N=186,590 (77.4%)
**Age at diabetes diagnosis, years, mean (SD)**	38 (19)	40 (19)	38 (19)
**Age group, n (%)**			
0-12	20,229 (8%)	3,400 (6%)	16,829 (9%)
13-17	17,790 (7%)	3,156 (6%)	14,634 (8%)
18-44	109,163 (45%)	25,921 (47%)	83,242 (45%)
45-64	71,905 (30%)	15,687 (29%)	56,218 (30%)
≥65	22,136 (9%)	6,469 (12%)	15,667 (8%)
**Sex, n (%)**			
Male	125,065 (52%)	28,299 (52%)	96,766 (52%)
Female	116,158 (48%)	26,334 (48%)	89,824 (48%)
**Race/ethnicity, n (%)**			
Hispanic	16,114 (7%)	3,621 (7%)	12,493 (7%)
Non-Hispanic Asian	3,530 (1%)	711 (1%)	2,819 (2%)
Non-Hispanic Black	29,620 (12%)	8,161 (15%)	21,459 (11%)
Non-Hispanic White	181,044 (75%)	39,197 (72%)	141,847 (76%)
Other/unknown[1]	10,915 (5%)	2,943 (5%)	7,972 (4%)
**Insurance type, n (%)**			
Commercial	156,231 (65%)	29,398 (54%)	126,833 (68%)
Medicaid	33,221 (14%)	7,983 (15%)	25,238 (14%)
Medicare	26,080 (11%)	6,573 (12%)	19,507 (10%)
Uninsured	17,474 (7%)	4,636 (8%)	12,838 (7%)
Unknown	8,217 (3%)	6,043 (11%)	2,174 (1%)

[1] Specific racial and ethnicity categories within the "other" group were not available in OLDW.

© 2025 Fang M et al. *JAMA Network Open*.

eTable 5. Characteristics of Youth and Adults With Type 1 Diabetes at the First Measurement of HbA_{1c} in the Study

Characteristics	Overall<br>N=186,590	Youth<br>N=26,853	Adults<br>N=159,737
**Age, years, mean (SD)**	40 (19)	12 (4)	45 (16)
**Age group, n (%)**			
0-12 year	13,915 (8%)	13,915 (52%)	-
13-17 year	12,938 (7%)	12,938 (48%)	-
18-44 year	80,513 (43%)	-	80,513 (51%)
45-64 year	58,025 (31%)	-	57,025 (36%)
65-88 year	21,199 (11%)	-	21,199 (13%)
**Sex, n (%)**			
Male	96,766 (52%)	14,060 (52%)	82,706 (52%)
Female	89,824 (48%)	12,793 (48%)	77,031 (48%)
**Race/ethnicity, n (%)**			
Hispanic	12,493 (7%)	2,687 (10%)	9,806 (6%)
Non-Hispanic Asian	2,819 (2%)	312 (1%)	2,507 (2%)
Non-Hispanic Black	21,459 (11%)	2,521 (9%)	18,938 (12%)
Non-Hispanic White	141,847 (76%)	19,822 (74%)	122,025 (76%)
Other/unknown[1]	7,972 (4%)	1,511 (6%)	6,461 (4%)
**Insurance type. n (%)**			
Commercial	118,483 (64%)	18,245 (68%)	100,238 (63%)
Medicaid	25,997 (14%)	6,758 (25%)	19,239 (12%)
Medicare	24,968 (13%)	-	24,968 (16%)
Uninsured	8,008 (4%)	866 (3%)	7,142 (4%)
Unknown	9,134 (5%)	984 (4%)	8,150 (5%)

[1] Specific racial and ethnicity categories within the “other” group were not available in OLDW.

© 2025 Fang M et al. *JAMA Network Open*.

eTable 6. Characteristics of Youth and Adults With Type 1 Diabetes at the First Measurement of HbA_{1c} by Year

	2009-2011		2012-2014		2015-2017		2018-2020		2021-2023	
	Youth N=5,988	Adults N=32,270	Youth N=12,012	Adults N=65,707	Youth N=14,282	Adults N=96,596	Youth N=11,097	Adults N=100,202	Youth N=6,500	Adults N=65,205
**Age, years**	12 (4)	44 (16)	12 (4)	45 (16)	12 (4)	45 (16)	12 (4)	46 (17)	12 (4)	48 (17)
**Age category, n (%)**										
0-12	2,950 (49%)		5,779 (48%)		6,398 (45%)		4,886 (44%)		2,829 (44%)	
13-17	3,038 (51%)		6,233 (52%)		7,884 (55%)		6,211 (56%)		3,671 (56%)	
18-44	-	16,440 (51%)	-	32,915 (50%)	-	47,215 (49%)	-	46,323 (46%)	-	28,124 (43%)
45-64	-	11,834 (37%)	-	24,417 (37%)	-	36,422 (38%)	-	38,022 (38%)	-	24,090 (37%)
65-88	-	3,996 (12%)	-	8,375 (13%)	-	12,959 (13%)	-	15,857 (16%)	-	12,991 (20%)
**Sex, n (%)**										
Male	3,103 (52%)	16,422 (51%)	6,216 (52%)	33,744 (51%)	7,346 (52%)	50,011 (52%)	5,725 (52%)	51,758 (52%)	3,375 (52%)	33,641 (52%)
Female	2,885 (48%)	15,848 (49%)	5,796 (48%)	31,963 (49%)	6,936 (49%)	46,585 (48%)	5,372 (48%)	48,444 (48%)	3,125 (48%)	31,564 (48%)
**Race/ ethnicity, n (%)**										
Hispanic	336 (6%)	1,408 (4%)	880 (7%)	2,921 (4%)	1,288 (9%)	4,875 (5%)	1,337 (12%)	6,038 (6%)	950 (15%)	4,872 (8%)
Non-Hispanic Asian	33 (1%)	268 (1%)	93 (1%)	659 (1%)	154 (1%)	1,157 (1%)	164 (2%)	1,514 (2%)	128 (2%)	1,374 (2%)
Non-Hispanic Black	388 (6%)	2,506 (8%)	1,006 (8%)	5,915 (9%)	1,314 (9%)	9,978 (10%)	975 (9%)	11,351 (11%)	659 (10%)	8,048 (12%)
Non-Hispanic White	4,955 (83%)	26,707 (83%)	9,491 (79%)	54,315 (83%)	10,832 (76%)	77,535 (81%)	7,914 (71%)	77,898 (78%)	4,340 (67%)	48,699 (75%)
Other/ unknown[1]	276 (4%)	1,381 (4%)	542 (5%)	1,897 (3%)	694 (5%)	3,051 (3%)	707 (6%)	3,401 (3%)	423 (6%)	2,212 (3%)
**Insurance type, n (%)**										
Commercial	4,097 (68%)	19,908 (62%)	8,421 (70%)	43,179 (66%)	9,968 (70%)	62,290 (65%)	7,851 (71%)	64,503 (64%)	4,590 (71%)	41,118 (63%)
Medicaid	1,406 (24%)	2,219 (7%)	2,949 (24%)	6,137 (9%)	3,445 (24%)	10,836 (11%)	2,726 (25%)	12,681 (13%)	1,662 (26%)	8,322 (13%)
Medicare	-	4,761 (15%)	-	10,604 (16%)	-	16,328 (17%)	-	18,672 (19%)	-	13,925 (21%)
Uninsured	124 (2%)	1,717 (5%)	406 (4%)	2,727 (4%)	522 (4%)	4,785 (5%)	316 (2%)	3,440 (3%)	91 (1%)	1,140 (2%)
Unknown	361 (6%)	3,665 (11%)	236 (2%)	3,060 (5%)	347 (2%)	2,357 (2%)	204 (2%)	906 (1%)	157 (2%)	700 (1%)

[1] Specific racial and ethnicity categories within the "other" group were not available in OLDW

© 2025 Fang M et al. *JAMA Network Open*.

eTable 7. Mean Hemoglobin A_{1c} in Youth and Adults With Type 1 Diabetes From 2009-2011 to 2021-2023

	2009-2011	2012-2014	2015-2017	2018-2020	2021-2023
Youth	N=5,988	N=12,012	N=14,282	N=11,097	N=6,500
Mean HbA1c, %	8.9 (1.5)	8.8 (1.6)	8.9 (1.6)	8.7 (1.7)	8.3 (1.7)
Adults	N=32,270	N=65,707	N=96,596	N=100,202	N=65,205
Mean HbA1c, %	8.2 (1.7)	8.3 (1.7)	8.4 (1.7)	8.3 (1.7)	8.0 (1.7)

Note: Standard deviations are indicated in paratheses.

© 2025 Fang M et al. *JAMA Network Open*.

eTable 8. Trends in Use of Continuous Glucose Monitoring and Insulin Pumps in Youths With Type 1 Diabetes From 2009-2011 to 2021-2023 by Race, Ethnicity, and Insurance Status

Non-Hispanic White						
Period	Commercial Insurance			Medicaid Insurance		
	CGM	Insulin pump	Both	CGM	Insulin pump	Both
2009-2011	4.3 (.3)	17.8 (.6)	1.4 (.2)	3.5 (.6)	16 (1.2)	1 (.3)
2012-2014	8.1 (.3)	23.5 (.5)	3.3 (.2)	4.9 (.5)	22.7 (1)	2.1 (.3)
2015-2017	22.6 (.5)	34.5 (.5)	11.6 (.4)	11.7 (.7)	32 (1)	5.8 (.5)
2018-2020	60.8 (.6)	42.4 (.6)	30.1 (.6)	44.1 (1.3)	36.5 (1.2)	19.8 (1)
2021-2023	84.1 (.6)	55.6 (.9)	52.3 (.9)	76.7 (1.4)	39.8 (1.6)	36.6 (1.6)
Non-Hispanic Black						
	Commercial Insurance			Medicaid Insurance		
Period	CGM	Insulin pump	Both	CGM	Insulin pump	Both
2009-2011	S	10.5 (2.5)	S	S	6.4 (1.7)	S
2012-2014	6.6 (1.1)	12.2 (1.4)	S	3.5 (.9)	13.7 (1.6)	S
2015-2017	10.8 (1.2)	16.1 (1.4)	3.7 (.7)	8.1 (1.2)	17.8 (1.6)	2.7 (.7)
2018-2020	46.3 (2.2)	27.6 (1.9)	19.1 (1.7)	28.3 (2.2)	23 (2.1)	8 (1.3)
2021-2023	79.5 (2.1)	39.7 (2.5)	38.6 (2.5)	73.2 (2.7)	30.9 (2.8)	30.6 (2.8)
Hispanic						
	Commercial Insurance			Medicaid Insurance		
Period	CGM	Insulin pump	Both	CGM	Insulin pump	Both
2009-2011	S	13.4 (2.7)	S	S	16.4 (3.5)	S
2012-2014	5.5 (1.1)	24.9 (2.1)	S	3.4 (.9)	15.4 (1.8)	S
2015-2017	16.8 (1.5)	28.1 (1.8)	10.5 (1.2)	8.4 (1.2)	15.4 (1.6)	3.4 (.8)
2018-2020	51.6 (1.9)	33.2 (1.8)	24.6 (1.7)	31.8 (2)	23.7 (1.8)	9.7 (1.3)
2021-2023	80.9 (1.8)	50.8 (2.2)	48 (2.2)	73.7 (2.3)	32.9 (2.5)	31.2 (2.4)

Note: Prevalence (with standard errors) are reported in each cell. Cells with counts <11 (indicated by an "S") were suppressed due to Optum Labs data policy.
Abbreviations: CGM = continuous glucose monitoring

© 2025 Fang M et al. *JAMA Network Open*.

eTable 9. Trends in Use of Continuous Glucose Monitoring and Insulin Pumps in Adults With Type 1 Diabetes From 2009-2011 to 2021-2023 by Race, Ethnicity, and Insurance Status

Non-Hispanic White									
Period	Commercial Insurance			Medicaid Insurance			Medicare Insurance		
	CGM	Insulin pump	Both	CGM	Insulin pump	Both	CGM	Insulin pump	Both
2009-2011	5.2 (.2)	13.5 (.3)	1.8 (.1)	4.1 (.5)	9.8 (.8)	1.1 (.3)	5.8 (.4)	10.6 (.5)	1.7 (.2)
2012-2014	9.6 (.2)	21 (.2)	4 (.1)	6.3 (.4)	15.3 (.6)	1.8 (.2)	7.5 (.3)	18 (.4)	2.8 (.2)
2015-2017	13.7 (.2)	29.2 (.2)	7.1 (.1)	9.7 (.3)	22 (.5)	4.3 (.2)	11 (.3)	24.9 (.4)	5 (.2)
2018-2020	39.3 (.2)	33.2 (.2)	17.8 (.2)	30.2 (.5)	25 (.5)	11.7 (.4)	32 (.4)	26.3 (.4)	12.7 (.3)
2021-2023	61.3 (.3)	35.4 (.3)	27.7 (.3)	50.9 (.7)	28.4 (.6)	21.3 (.6)	52.5 (.5)	27.4 (.4)	19.8 (.4)
Non-Hispanic Black									
	Commercial Insurance			Medicaid Insurance			Medicare Insurance		
Period	CGM	Insulin pump	Both	CGM	Insulin pump	Both	CGM	Insulin pump	Both
2009-2011	3.7 (.6)	5.1 (.6)	S	S	6.5 (1.2)	S	5.6 (1.1)	3.4 (.9)	S
2012-2014	7 (.5)	10.1 (.5)	1.8 (.2)	6.8 (.7)	7.9 (.8)	S	8.7 (.9)	8.2 (.8)	1.6 (.4)
2015-2017	10.1 (.4)	13.5 (.5)	3.6 (.3)	9.2 (.6)	12.1 (.7)	2.4 (.3)	9.1 (.7)	13.2 (.8)	3.1 (.4)
2018-2020	29 (.6)	14.4 (.5)	7.6 (.3)	24 (.8)	12 (.6)	5.2 (.4)	23.4 (.9)	12.6 (.7)	5.6 (.5)
2021-2023	53.4 (.7)	15.3 (.5)	12.9 (.5)	42.5 (1.2)	13.6 (.8)	9.4 (.7)	45.9 (1.3)	15.3 (.9)	10.9 (.8)
Hispanic									
	Commercial Insurance			Medicaid Insurance			Medicare Insurance		
Period	CGM	Insulin pump	Both	CGM	Insulin pump	Both	CGM	Insulin pump	Both
2009-2011	3.3 (.8)	9.4 (1.2)	S	7.3 (1.9)	S	S	9.2 (2.5)	8.4 (2.4)	S
2012-2014	9.1 (.7)	16.4 (1)	3.6 (.5)	6.7 (1.2)	12.8 (1.5)	S	9.8 (1.7)	11.1 (1.8)	3.6 (1.1)
2015-2017	10.6 (.6)	17.4 (.8)	4.6 (.4)	10.1 (1)	11.9 (1.1)	2.3 (.5)	10.3 (1.2)	13.1 (1.3)	2.3 (.6)
2018-2020	34.7 (.8)	19.3 (.7)	11.8 (.6)	28.1 (1.3)	15 (1)	8.2 (.8)	26.4 (1.5)	13.1 (1.1)	6.3 (.8)
2021-2023	58.2 (1)	22.7 (.8)	19 (.8)	56.5 (1.4)	21 (1.2)	17 (1.1)	52.6 (1.8)	19.4 (1.4)	15.5 (1.3)

Note: Prevalence (with standard errors) are reported in each cell. Cells with counts <11 (indicated by an "S") were suppressed due to Optum Labs data policy.
Abbreviations: CGM = continuous glucose monitoring

© 2025 Fang M et al. *JAMA Network Open*.
