# Supplementary material for: Trends and Disparities in Technology Use and Glycemic Control in Type 1 Diabetes
Source: JAMA Netw Open. 2025 Aug 11;8(8):e2526353. doi: 10.1001/jamanetworkopen.2025.26353 (PMC12340658; doi:10.1001/jamanetworkopen.2025.26353)
Supplement: Supplement 2. — Data Sharing Statement [file jamanetwopen-e2526353-s002.pdf]

Data Sharing Statement

Fang. Trends and Disparities in Technology Use and Glycemic Control in Type 1 Diabetes. *JAMA Netw Open*. Published August 11, 2025. doi:10.1001/jamanetworkopen.2025.26353

Data
Data available: No
